# Improving Research into Models of Maternity Care to Inform Decision Making

**DOI:** 10.1371/journal.pmed.1002135

**Published:** 2016-09-27

**Authors:** Ank de Jonge, Jane Sandall

**Affiliations:** 1 Midwifery Science, AVAG and the EMGO Institute for Health and Care Research, VU University Medical Center, Amsterdam, The Netherlands; 2 Women’s Health Academic Centre, Division of Women’s Health, Faculty of Life Sciences & Medicine, King’s College London, London, United Kingdom

## Abstract

In a Perspective, Ank de Jonge and Jane Sandall discuss research on models of maternity care led by midwives.

The key aim of health services research is to inform decisions made by service users, health care providers, and policy makers. Therefore, the evidence produced needs to be robust, and the limitations clearly described. Maternity care is one area of health services that needs strong evidence to underpin the way it is organised.

There is a growing interest across the world in midwife-led continuity of care (MLCC) for childbearing women and their babies [1,2]. In MLCC, one midwife or a small team of midwives lead the planning, organisation, and delivery of care, with some care delivered in consultation with obstetric staff in a timely and appropriate process [1]. In a Cochrane systematic review of 15 trials involving 17,674 women, MLCC compared favourably with obstetrician-provided or family doctor-provided care or shared models of care [1]. For example, women who had MLCC were less likely to have a preterm birth before 37 weeks (average risk ratio [RR] 0.76, 95% confidence interval [CI] 0.64–0.91), foetal loss/neonatal death before 24 weeks (average RR 0.81, 95% CI 0.67–0.98), regional analgesia (average RR 0.85, 95% CI 0.78–0.92), instrumental vaginal birth (average RR 0.90, 95% CI 0.83–0.97), and episiotomy (average RR 0.84, 95% CI 0.77–0.92). There were no significant differences in caesarean section, foetal loss or neonatal death at or after 24 weeks gestation, low Apgar scores, or rates of neonatal convulsions or admission to a neonatal intensive care unit. Overall, more women were satisfied with MLCC, and there was a trend towards a cost-saving effect compared to other models of care. Based on these positive results, MLCC has been advocated as an effective way to organise maternal and newborn care [2,3].

## MLCC in New Zealand

So far, New Zealand is the only high-income jurisdiction where MLCC has been implemented throughout the country. Therefore, this country is ideally suited to perform large-scale cohort studies into MLCC to examine rare, severe adverse outcomes. In a research article in *PLOS Medicine*, Wernham et al. asked an important research question about the safety of MLCC and performed a large study over a 5-year period using routinely collected national data and compared 20,662 women who had an obstetrician or general practitioner to 223,385 women who had a midwife as their lead maternity carer at first registration [4]. Babies born to women with an obstetrician or general practitioner as their lead maternity carer had a significantly lower rate of Apgar scores of less than seven at 5 minutes (adjusted odds ratio [adj OR] 0.52, 95% CI 0.43–0.64), birth-related asphyxia (adj OR 0.45, 95% CI 0.32 to 0.62), and neonatal encephalopathy (adj OR 0.61, 95% CI 0.38–0.97). No significant differences were found in perinatal mortality before the completion of the 27th day of life.

How can we explain the striking differences in the findings of Wernham et al.’s study as compared with those of the Cochrane review? The number of inclusions was much larger in Wernham et al.’s study, and therefore, differences in relatively rare outcomes could have been detected more easily than in the Cochrane review. In addition, the quality of care offered to women with a midwife versus an obstetrician as the lead maternity carer at first entry in New Zealand may be different. However, known and unknown confounding factors could also affect the findings of an observational study [5]. Firstly, in Wernham et al.’s study, only infant outcomes of pregnancies equal to or greater than 37 weeks were reported, even though other outcomes for mothers and infants should have been available. This selective reporting of only a few outcomes is not helpful in providing an overall view of risks and benefits. Medical interventions can lead to serious morbidity for women [6] and the authors themselves acknowledge that their findings should be interpreted in the context of benefits of midwife-led care such as lower intervention rates [1]. It is time to develop consensus on the design of observational studies and a core outcome dataset of maternal and infant outcomes for research into models of maternity care so that future studies will provide the full information that is required for decision making. Given the fact that infant outcomes differ considerably between nulliparous and multiparous women, it would be helpful to analyse results for these subgroups separately rather than controlling for parity as a confounder [6].

Secondly, in research on complex interventions, it is important to describe in detail the model of care being provided. Wernham et al. compared women based on their lead maternity carer at first registration in pregnancy, but it was not possible to report what model of care was received at the onset of labour. All outcomes, apart from stillbirth, were labour related. To evaluate outcomes of intrapartum care, the appropriate comparison would be women in midwife-led care versus medical-led care at the onset of labour [6,7]. Despite the fact that only 1.9% of women in the midwife-led group in Wernham et al.’s study were registered with a hospital or obstetrician at the time of birth, many more may have had medical input from the onset of labour in a shared care arrangement, and women in the medical-led group were also likely to receive midwifery support. Even so, the study results raise the important question of why more adverse infant outcomes were found among women who had a midwife as their lead maternity carer at first registration.

Thirdly, it is not possible to draw causal relationships from observational studies. Although Wernham et al. controlled for several confounding factors, residual differences between the groups may have remained. Differences in the type of women who choose a midwife or an obstetrician or general practitioner as their lead maternity carer are likely to be important confounders. The authors did not control for place of birth or distance to hospital. Most care in rural areas is provided by midwives. About 16% of women in New Zealand give birth in one of the 48 primary maternity units in which midwives provide care; 32 of these are more than an hour’s drive from an obstetric hospital [8]. In a Dutch study, adverse perinatal outcome rates were higher among women who lived more than 20 minutes from a hospital [9].

Nevertheless, even though transfer from a rural area to an obstetric unit, if required, may be challenging [8], there are social and economic arguments for maintaining rural maternity services [10]. Many women in rural areas prefer to give birth in their own community, and relocating near an obstetric hospital prior to birth comes at a high financial and social cost to rural women [10]. In addition, if care is centralised in obstetric units, more women may give birth before arrival at a hospital, and unplanned out-of-hospital births are associated with higher rates of adverse perinatal outcomes [11]. In a questionnaire study among low-risk women in the Christchurch area, 1 in 31 women who planned birth in a primary unit and 1 in 95 women who planned birth in a maternity hospital had an unplanned home birth [12].

Exploring cases of adverse outcomes in more depth in perinatal audit studies can provide insight into other factors that could explain higher rates of adverse outcomes in MLCC. This may highlight aspects of clinical care, collaboration between professionals, and organisation of care that can be improved.

## Closer Examination of Midwife-Led and Medical-Led Care

There are variations in the way MLCC is organised: it may be offered only to low-risk women or to women with moderate risks as well, there may or may not be routine medical input, and birth may take place in obstetric units but also in midwife-led units and at home [1]. For example, in the Netherlands, MLCC is only offered to low-risk women who are referred to obstetrician-led care if they need interventions such as continuous foetal monitoring or augmentation [6]. In New Zealand, on the other hand, most women remain in MLCC if they require medical interventions, although midwives may consult with obstetricians [13]. Multidisciplinary research is required to understand mechanisms leading to differences in processes, outcomes, costs, and women’s experiences between MLCC and other models of care. This research should unpack the effects of different elements of MLCC such as the midwife as the lead carer, the process of risk assessment, the philosophy of care, continuity of care, and place of birth. High-quality studies are characterised by a clear description of the models of care, comparison of appropriate groups, the use of a core outcome dataset for quantitative studies that includes key outcomes for mother and infant, adjustment for potential confounders, and analysis of key subgroups such as parity.

## Abbreviations

CI: confidence interval
MLCC: midwife-led continuity of care
OR: odds ratio
RR: risk ratio
